# Sleep patterns in children with ADHD: a population-based cohort study from birth to 11 years

**DOI:** 10.1111/j.1365-2869.2012.01054.x

**Published:** 2012-10-12

**Authors:** Nicola Scott, Peter S Blair, Alan M Emond, Peter J Fleming, Joanna S Humphreys, John Henderson, Paul Gringras

**Affiliations:** 1 Guy’s and St Thomas’ NHS Foundation TrustLondon, UK; 2 School of Social and Community Medicine University of BristolBristol, UK; 3 King’s College London and Guy’s and St Thomas’ NHS Foundation TrustLondon, UK

**Keywords:** attention deficit hyperactivity disorder, Avon Longitudinal Study of Parents and Children, sleep

## Abstract

Associations between sleep duration and disturbance in infancy and early childhood and attention deficit hyperactivity disorder diagnoses were investigated. Data from the Avon Longitudinal Study of Parents and Children, a population-based prospective longitudinal birth-cohort study of children born in 1991–1992 in South-West England, were employed. Eight thousand, one hundred and ninety-five children were assessed using the Development and Well-Being Assessment. One hundred and seventy-three cases (2.1%) met criteria for attention deficit hyperactivity disorder. Parental report at eight time points showed children with attention deficit hyperactivity disorder slept less than peers. Absolute differences were small and mainly restricted to night-time sleep, with no strong evidence of differences from controls, except at 69 months {5 years 9 months; 12 min (95% CI: 5–19), *P* = 0.001}, at 81 months {6 years 9 months; 15 min (95% CI: 8–22), *P* < 0.001} and at 115 months {9 years 7 months; 11 min (95% CI: 4–18), *P* = 0.001}. The attention deficit hyperactivity disorder group had more night-waking at every age, significant from about 5 years. When tracking children’s sleep along a normative centiles chart, a shift in sleep duration from one centile to a lower centile was a useful predictor of attention deficit hyperactivity disorder. Age-specific decreases of >1SD in sleep duration across adjacent time points was a significant predictor of attention deficit hyperactivity disorder at 3–5 years (*P* = 0.047). In children with attention deficit hyperactivity disorder, shorter sleep duration and sleep disturbances appear early and predate the usual age of clinical diagnosis. The rate of change of sleep duration relative to an individual, rather than absolute sleep duration at any stage, may prove beneficial in identifying increased risk of attention deficit hyperactivity disorder.

## Introduction

A number of associations between sleep problems and attention deficit hyperactivity disorder (ADHD) have been described (Cortese *et al*., 2009; Sadeh *et al*., 2006). These include sleep-onset problems, sleep phase delay syndrome, increased movements in sleep, daytime sleepiness and altered total sleep time (Corkum *et al*., 1998, 1999; Cortese *et al*., 2006; Konofal *et al*., 2001, 2010; Mayes *et al*., 2009; see Cortese *et al*., 2009; Sadeh *et al*., 2006 for reviews).

However, many of the studies come from cross-sectional studies of school-age children with limited controlling for confounding or consideration of the longitudinal sleep trajectory. Interpretations of these associations include poor sleep quality or quantity being part of a causal mechanism of ADHD, ADHD and its treatments causing sleep problems, or that the two share a common aetiology (Gringras *et al*., 2007).

Prospective cohort studies offer the advantage that developmental trajectories of sleep and their temporal relationship to the onset of ADHD symptoms can be determined, and thus offer more insight about possible direction of causation. Thunstrom (2002) reported a 3-year follow-up study of 27 children with chronic sleep problems, of which seven were subsequently diagnosed with ADHD symptoms at age 5.5 years. Touchette *et al*. (2007) reported from the Quebec Longitudinal Study of Child Development (Canada) that sleep duration of less than 10 h per night, especially before the age of 41 months, was associated with hyperactivity/impulsivity symptoms and lower cognitive functioning at age 6 years. Cohort studies to date have predominantly considered pre-school sleep patterns, and relied on screening questionnaires for hyperactive symptoms, rather than standardised psychiatric measures for ADHD.

We used prospectively collected data from the Avon Longitudinal Study of Parents and Children (ALSPAC) to investigate sleep patterns and trajectories from 6 months after birth to 11 years old, and their relation to ADHD diagnoses established by standardised psychiatric interviews according to DSM-IV criteria (American Psychiatric Association., 2000).

## Materials and Methods

### Description of sample

The ALSPAC is a longitudinal cohort study following the health and development of children who had an expected date of delivery between April 1991 and December 1992, and were resident in the Avon area of South-West England at the time of their birth. Fourteen thousand, five hundred and forty-one mothers enrolled in pregnancy, resulting in 14 062 live births, of whom 13 988 survived the first year. Full details of the questionnaires used, the biological samples retained, the examinations and observations of the children are available on the ALSPAC website (www.bristol.ac.uk/alspac). The study was approved by the Local Research Ethics Committee and by the ALSPAC Ethics and Law Committee. Data were collected and collated at the University of Bristol, School of Social and Community Medicine. Further information on ethical approval and procedures around informed consent are available on the ALSPAC website.

### ADHD Diagnosis

The Development and Well-Being Assessment (DAWBA- www.DAWBA.com; Goodman *et al*., 2000) is a package of interviews, questionnaires and rating techniques designed to generate ICD-10 (World Health Organisation., 1992) and DSM-IV (American Psychiatric Association., 2000) psychiatric diagnoses on 5–17 years olds. For ADHD, the predictive value of a positive or negative DAWBA (Goodman *et al*., 2000) diagnosis is greater than 0.8, with negligible bias. The DAWBA allows measurement of duration and impact of symptoms, through the use of both closed and open-ended questions, a computerised set of risk probability bands, and finally the opinion of an experienced trained clinical rater. The DAWBA was administered to the ALSPAC cohort at about age 7 years (range 90–111 months).

Development and Well-Being Assessment questionnaires were completed on 8253 children by their parents. Of these, 4008 also had a partially or fully completed teacher questionnaire. The questionnaires of those with symptoms of ADHD were then rated by an experienced child psychiatrist to confirm DSM-IV (American Psychiatric Association., 2000) diagnoses. Because the symptoms of ADHD can sometimes be mimicked by anxiety or affective disorders, the clinical rater reviewed emotional symptoms carefully before assigning ADHD diagnoses. Under DSM-IV (American Psychiatric Association., 2000) rules, a diagnosis of ADHD is ruled out by a coexistent pervasive developmental disorder. Consequently, all questionnaires and transcripts were carefully screened, and 31 children were excluded from the subsequent analyses. An additional 17 children were not sent or did not receive questionnaires and, therefore, the denominator for this analysis is 8195 children.

### Sleep measures

Parental questionnaires at 6, 18, 42, 69, 81, 115 and 140 months old asked detailed questions about children’s sleep patterns. Sleep duration was calculated from the time children ‘normally’ went to bed and woke in the morning and an estimated number of hours slept during the day (none, <1, 1–2 or >2 h). Night-time waking events were categorised into none, 1, 2 or 3 or more. Age windows were used for each questionnaire to ensure data were from the child at the appropriate age. A more detailed account of the sleep data collection methodology and analysis has recently been published (Blair *et al*., 2012).

### Other variables measured

Covariates potentially associated with sleep or ADHD were tested. As the families in this cohort were predominantly white, the remaining ethnic groups, the largest of which are Black Caribbean and Asian, have been grouped together as a non-white minority. Socio-economic status was categorised using the highest educational qualification of the mother and paternal occupational classification obtained at the beginning of the study. These were dichotomised using the cut-offs of those mothers who achieved a school-leaving certificate below the standard level (GCSE grade C or equivalent) expected at age 16 years or no qualifications and those partners whose occupation was classified as IV/V (semi-skilled or unskilled) according to the Registrar General’s occupational classification (Office of Population Censuses and Surveys., 1980). Pre-term infants were defined as those born before 37 weeks gestation, and low birth weight as those with birth weight below 2500 g. Young mothers were defined as those aged less than 21 years when the study subject was born, and older mothers as those aged more than 35 years. Large families were defined as those where the study child had three or more older siblings.

Where we have focussed on specific age groups we define ‘infancy’ as birth to 1 year, the ‘pre-school years’ as 1–4 years old, and the ‘primary school years’ as aged 5–11 years.

### Statistical analysis

Data manipulation and analysis was performed using the spss (version 19.0.0.1; SPSS windows; Chicago, IL, USA) statistical program. For the univariable analysis the chi-squared test was utilised to test differences in proportions and Fisher’s exact test when an expected cell was less than 5. Multivariable linear regression modelling was conducted using the sleep duration as the dependent variable and controlling for factors significantly associated with ADHD or sleep duration. Statistical significance was measured at the 5% level and the Backward Step variable selection was used. Standardised residuals were plotted against predicted values and Cook’s distances plotted to measure influential observations.

Rates of falls in sleep duration across normative centiles were analysed by constructing *z*-scores for sleep duration and then considering the proportion of children at each time point who decreased sleep duration by more than 1SD. Multivariable logistic regression modelling was conducted controlling for factors significantly associated with ADHD.

## Results

### Ascertainment

In total there were 173 ADHD cases (2.1%), of which 70 were classified as ‘inattentive’ ADHD (40.5%), 24 classified as ‘hyperactive-impulsive’ ADHD (13.9%), and a further 79 children had a combination of the two types of disorder (45.7%). It was not possible to obtain parent and teacher reports on everyone, with 96 (55%) including both and 77 (45%) being based on DAWBA interview of parent alone. In this paper we present results for all 173 children with ADHD, compared with the remaining 8022 children in the cohort who underwent DAWBA assessment at aged 7 years.

### Demographic profile of ADHD cases

Children with ADHD differed from subjects without ADHD in a number of variables (Table 1).

**Table 1:** Demographic profile of ADHD cases

*Variable*	*Category*	*ADHD* n/N (%)	*Rest of cohort* n/N (%)	P-value*
Gender	Male	146/173 (84.4%)	4059/8022 (50.6%)	<0.001
Gestation	<37 weeks	18/173 (10.4%)	398/7624 (5.2%)	0.001
Birth weight	<2500 g	13/173 (7.5%)	350/7930 (4.4%)	0.051
Maternal age	21–35 years	148/173 (85.5%)	7069/8022 (88.1%)	Ref. group
	<21 years	12/173 (6.9%)	285/8022 (3.6%)	0.02
	>35 years	13/173 (7.5%)	668/8022 (8.3%)	0.80
Paternal social class	IV or V	19/141 (13.5%)	763/7141 (10.7%)	0.29
Maternal qualifications	Less than standard level at 16 years†	23/163 (14.1%)	1119/7792 (14.4%)	0.52
Ethnicity	Non-white	9/159 (5.7%)	291/7667 (3.8%)	0.23
Siblings	Three or more	9/163 (5.5%)	361/7779 (4.6%)	0.60

ADHD, attention deficit hyperactivity disorder.

* Chi-square test.

† Below GCSE grade C.

Although ADHD diagnosis was not more common amongst children of deprived families, from larger families, those of non-white ethnicity or amongst older mothers, there were significant univariable associations with young maternal age, pre-term birth and male sex. Birth weight was not a significant factor, but was noted to be very close to significant (*P* = 0.051). Male sex was consistently associated with inattentive (80% male), hyperactive-impulsive (79% male) and combined (90% male) ADHD types.

### Sleep and ADHD cases

#### Ascertainment of sleep data

Of 8195 children assessed using DAWBA, sleep data were available for about 90% at each age point prior to the ADHD assessment (7 years), and slightly less for the two subsequent time points.

#### Night-time sleep duration

Children with ADHD slept on average 13 min less than the rest of the cohort at night during infancy (Table 2), and continued to have shorter sleep during the pre-school years, although the difference was less marked. The difference again became apparent and statistically significant during primary school, but was less marked by secondary school age (11 years).

**Table 2:** Night-time sleep duration for ADHD cases and rest of cohort

	*ADHD cases*			*Rest of cohort*			
*Time point*	N	*Mean*	*SD*	N	*Mean*	*SD*	P-value*
6 months	164	10 h 35 min	1 h 24 min	7573	10 h 48 min	1 h 19 min	0.04
18 months (1 year 6 months)	156	11 h 16 min	1 h 09 min	7431	11 h 19 min	1 h 02 min	0.50
30 months (2 years 6 months)	143	11 h 08 min	1 h 04 min	6986	11 h 14 min	0 h 58 min	0.20
42 months (3 years 6 months)	147	11 h 13 min	1 h 0 min	7143	11 h 16 min	0 h 52 min	0.47
69 months (5 years 9 months)	146	11 h 0 min	0 h 56 min	7056	11 h 17 min	0 h 41 min	<0.001
81 months (6 years 9 months)	143	10 h 50 min	0 h 51 min	7048	11 h 08 min	0 h 40 min	<0.001
115 months (9 years 7 months)	137	10 h 13 min	0 h 48 min	6741	10 h 27 min	0 h 39 min	<0.001
140 months (11 years 8 months)	126	9 h 44 min	0 h 50 min	6164	9 h 49 min	0 h 38 min	0.19

ADHD, attention deficit hyperactivity disorder.

* *t*-test.

Children with diagnosed ADHD consistently went to bed later than the rest of the cohort across most ages, although the average difference rarely exceeded 10 min. They also tended to wake earlier by a few minutes (Table 3).

**Table 3:** Bedtimes and wake-times of ADHD cases and rest of cohort

	*ADHD cases*			*Rest of cohort*		
*Time point (months)*	N	*Bedtime (hours)*	*Wake-time (hours)*	N	*Bedtime (hours)*	*Wake-time (hours)*
6	163	20:17	06:53	7541	20:07	06:55
18	156	19:49	07:04	7431	19:45	07:04
30	143	19:50	06:57	6986	19:49	07:03
42	147	19:45	06:58	7143	19:45	07:01
69	146	20:04	07:04	7056	19:52	07:09
81	143	20:15	07:05	7048	20:04	07:12
115	137	21:02	07:15	6741	20:51	07:18
140	126	21:27	07:11	6164	21:22	07:10

ADHD, attention deficit hyperactivity disorder.

#### Night-time wakings

The proportion of children who woke three or more times during the night was higher amongst the children with ADHD at every time point, but was not significant in infancy or amongst pre-school children. From 5 years old, frequent waking was negligible (<1%) amongst the rest of the cohort, but began to increase amongst children diagnosed with ADHD from 3% at 5 years to 6% at 11 years (Table 4).

**Table 4:** Night-time waking for ADHD cases and rest of cohort (three or more times during the night)

*Time point (months)*	*ADHD cases* n/N (%)	*Rest of cohort* n/N (%)	P-*value*
6	22/163 (13.5)	768/7518 (10.2)	0.17
18	15/152 (9.9)	476/7303 (6.5)	0.10
30	8/140 (5.7)	346/6921 (5.0)	0.70
42	4/145 (2.8)	180/7093 (2.5)	0.79*
69	4/142 (2.8)	42/6866 (0.6)	0.01*
81	5/141 (3.5)	30/6898 (0.4)	0.001*
115	8/125 (6.4)	22/6411 (0.3)	<0.001*

ADHD, attention deficit hyperactivity disorder.

* Fisher’s exact test.

#### Daytime sleep duration

Children with ADHD had marginally shorter reported daytime sleep in early childhood and slightly longer in later childhood (Table 5). Restricting the analysis to children with information on daytime sleep duration for all six time points; 45.8% (49/107) of children with ADHD had stopped daytime sleep after 18 months, a slightly higher proportion than the rest of the cohort (40.0%; 2119/5294), but not statistically significant (*P* = 0.23).

**Table 5:** Daytime sleep duration for ADHD cases and rest of cohort

	*ADHD cases*			*Rest of cohort*			
*Time point (months)*	N	*Mean*	*SD*	N	*Mean*	*SD*	P-value*
6	164	2 h 20 min	1 h 08 min	7573	2 h 21 min	1 h 03 min	0.81
18	156	1 h 22 min	0 h 38 min	7431	1 h 27 min	0 h 32 min	0.04
30	143	0 h 41 min	0 h 44 min	6986	0 h 43 min	0 h 44 min	0.55
42	147	0 h 15 min	0 h 33 min	7143	0 h 14 min	0 h 31 min	0.80
69	146	0 h 3 min	0 h 16 min	7056	0 h 01 min	0 h 09 min	0.03
81	143	0 h 1 min	0 h 13 min	7048	0 h 0.5 min	0 h 07 min	0.18

ADHD, attention deficit hyperactivity disorder.

* *t*-test.

#### Total sleep duration

At every evaluation, children with ADHD and related sub-types had decreased sleep duration compared with the rest of the cohort, but this was more marked and only statistically significant during the younger primary school years (Table 6).

**Table 6:** Total sleep duration for ADHD cases and rest of cohort

	*ADHD cases*			*Rest of cohort*			
*Time point (months)*	N	*Mean*	*SD*	N	*Mean*	*SD*	P-value*
6	164	12 h 55 min	1 h 49 min	7573	13 h 09 min	1 h 39 min	0.07
18	156	12 h 37 min	1 h 17 min	7431	12 h 46 min	1 h 17 min	0.13
30	143	11 h 48 min	1 h 05 min	6986	11 h 57 min	1 h 03 min	0.11
42	147	11 h 27 min	0 h 59 min	7143	11 h 30 min	0 h 54 min	0.58
69	146	11 h 03 min	0 h 55 min	7056	11 h 18 min	0 h 41 min	<0.001
81	143	10 h 51 min	0 h 53 min	7048	11 h 09 min	0 h 40 min	<0.001
115	137	10 h 13 min	0 h 48 min	6741	10 h 27 min	0 h 39 min	<0.001
140	126	9 h 44 min	0 h 50 min	6164	9 h 49 min	0 h 38 min	0.19

ADHD, attention deficit hyperactivity disorder.

* *t*-test.

Using a multivariable linear regression model and adjusting for variables associated with both sleep duration and ADHD, the total sleep duration was significantly less for ADHD children at 69 months (by 12 min), 81 months (by 15 min) and 115 months (by 11 min; Table 7).

**Table 7:** Multivariate analysis of total sleep duration for ADHD cases and rest of cohort

*Time point (months)*	N *in model*	B-*value*	*Minutes of less sleep (95% CI)*	P-*value*
69	7021	0.198	12 (5–19)	0.001
81	7002	0.245	15 (8–22)	<0.001
115	6127	0.183	11 (4–18)	0.001

Adjusting for gender, maternal age (both younger and older mothers), pre-term births and larger families (three or more children per family). Cook’s distances identified one influential observation that was dropped from the above models.

### Tracking sleep changes across sleep centiles

**Figure 1:**
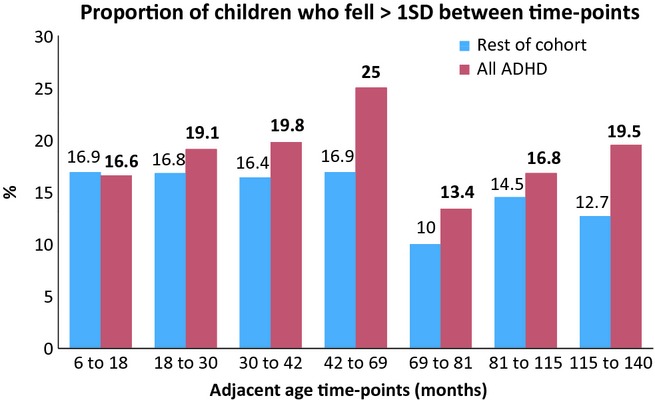
Proportion of children who fell between >1SD between time points. ADHD, attention deficit hyperactivity disorder.

Comparing children who decreased sleep duration between two time points by more than 1SD, there was a greater proportion of children with ADHD at each time point except between 6 and 18 months (Fig. 1). This was significant (Chi-square test: *P* = 0.01) between 42 and 69 months, when 34/136 children with ADHD (25.0%) had a substantial fall in sleep duration compared with 1100/6528 (16.9%) making up the rest of the cohort. Between 42 and 69 months the mean fall in sleep duration in the overall cohort was only 13 min between these ages, but the fall of 1SD constitutes a further 71 min decrease. In a logistic regression model this fall of more than 1SD remained a significant predictor of ADHD {*P* = 0.047, OR = 1.49 (1.01–2.22)} after controlling for gender, maternal age and gestational age. There was also a significant difference (Chi-square test: *P* = 0.03) found between 115 and 140 months, when 22/113 children with ADHD (19.5%) had a substantial fall in sleep duration compared with 732/5767 (12.7%) of the rest of the cohort. In a logistic regression model this fall of more than 1SD remained a significant predictor {*P* = 0.008, OR = 1.93 (1.19–3.12)} after again controlling for gender, maternal age and gestational age.

## Discussion

In all measures of sleep duration and continuity children with ADHD slept for a shorter time, and woke more than their peers. The main reason for their shorter sleep durations was later bedtimes. The effect sizes were very small, little strong evidence of cross-sectional differences from controls at most time points. The age when children diagnosed with ADHD slept significantly less than their peers seems to be about 5–9 years old. Although children with ADHD slept less than their peers, and stopped requiring a daytime sleep at an earlier age than their peers, we did not find strong evidence of differences in daytime sleep duration. This finding does not contradict the objective evidence reported of increased sleepiness on formal multiple sleep latency testing, which measures sleep pressure during carefully confined conditions, rather than actual daytime sleep duration (Cortese *et al*., 2006).

A different strategy, adopted in many other clinical domains, of identifying falls across normative centiles in longitudinal data more commonly predated the diagnosis of ADHD, and we suggest this approach warrants exploration in future studies. We found that considering a 1SD fall in *z*-scores for sleep duration between two adjacent time points was a significant predictor in identifying children later diagnosed with ADHD for six of the seven adjacent time points.

We have previously shown that although children’s sleep duration tracks their early sleep duration centiles over short periods of study, this relationship steadily weakens over time (Blair *et al*., 2012). Thus, as well as a remarkable natural variation in sleep duration throughout childhood, individual children do not remain ‘short’ or ‘long’ sleepers throughout childhood, but ‘move’ between centiles. We suspect that this accounts for the relatively small differences in sleep durations when looking at cross-sectional, absolute sleep durations at every age. However, when measuring changes relative to the individual, the magnitude of falls in sleep duration are more striking clinically. For example, between the ages of 42 and 69 months, a 1SD fall represents a reduction in sleep duration of more than 1 h and 20 min.

The strength of this study is that the DAWBA is a validated diagnostic tool used at a late enough age to allow a confident diagnosis of ADHD. Unlike other studies we did not rely on screening measures for ADHD traits at a young age that arguably may be conflated with sleep problems, or even represent sub-threshold and clinically normal behaviours rather than true ADHD.

There are a number of limitations that need consideration in this study. Although the parent DAWBA interview enquires about behaviours in schools and other environments, ideally there would have been parallel DAWBA teacher assessments on all the children. However, there were practical difficulties in encouraging all the teachers to complete a standardised assessment within a limited time window. Data from a UK population-based study that employed the DAWBA suggest that this potentially results in an underestimate of the prevalence of ADHD (Ford *et al*., 2003). Thus, we might have underestimated the numbers of children with ADHD, although in fact our overall ADHD prevalence (2.1%) is not out of keeping from that reported from the UK 1999 survey (2.23%) of over 10 000 children (Ford *et al*., 2003).

We were also reliant on subjective (parental reported) measures of sleep patterns and sleep behaviours. There are often differences between sleep difficulties reported by parents and those shown on objective sleep measures (Cortese *et al*., 2009). This study did collect contemporary sleep reports, reducing likelihood of any recall bias, and had a large enough sample size to allow comparisons with typically developing peers. For a study of this size, objective assessments over time are extremely difficult. In the paper (Blair *et al*., 2012) we have discussed the limitations of relying on subjective sleep data, but also why the data are still helpful and reliable. In this particular case the subjective nature of the data means that although children with ADHD are reported as sleeping less, this does not take into account their actual, objective sleep efficiency. Total sleep duration was calculated from parental reports of estimated bedtime, wake-time and daytime naps. This did not account for sleep latency, as an estimated bedtime did not differentiate between when the child goes to bed and goes to sleep. Also, night-time sleep duration did not take into account duration of any waking events. If one extrapolates from polysomnographic and actigraphy data, we feel it is likely that the reduced sleep duration and increased night waking reported is likely to be subjectively and objectively real.

A limitation of most longitudinal studies conducted over several years is that missing data and loss to follow-up are more likely in the most socioeconomically deprived groups. The ALSPAC study is no different, but of sufficient size that although some of the vulnerable groups were lost through this attrition, enough families remained in the study to differentiate between even relatively small social groupings.

We have not addressed whether psychosocial problems in the family might be associated with poor sleep and ADHD in children (Thunstrom, 2002). Dorris *et al*. (2008) suggest that it is important to consider parental limit setting and contingency management when assessing and managing sleep problems in children with ADHD. It is important to note, however, that in a study considering sleep hygiene and bedtime routines, these were equally well implemented for children with ADHD as for other children (Van der Heijden *et al*., 2006). In a review of 22 longitudinal studies, Hemmi *et al*. (2011) concluded that regulatory problems in infancy, including sleep, were associated with externalising behaviours and ADHD.

We do not have accurate information on which children were prescribed stimulant or other medication for their ADHD. At the time of the study it is very unlikely in the UK that medication treatment with stimulants would have commenced before 6 years old. The UK has historically treated far fewer children with ADHD than other countries. In 1995, for example, only 0.03% of children in the UK were on medication for ADHD in contrast to 3% in the USA and 1.7% in Australia (Parliamentary Office of Science and Technology., 1997). Mayes *et al*. (2009) and others have noted that medicated children had greater difficulty falling asleep than unmedicated children, although they suggest the medication prescription might be a proxy marker for ADHD severity. This uncontrolled factor might therefore have influenced the older children with ADHD in the cohort.

Comorbidities commonly associated with ADHD include anxiety, depression oppositional defiant disorder and autistic spectrum disorders (ASD; Faraone *et al*., 1998; Greene *et al*., 2002; Jensen *et al*., 1993; Wilens *et al*., 2002), but this study is underpowered to consider the impact of comorbidities in ADHD on sleep disturbance. The exclusion of children with ASD from DSM-IV (American Psychiatric Association., 2000) ADHD is arguably a weakness of this version of the diagnostic classification system, as in clinical practice the two are often comorbid. Our data on sleep trajectories in autism will be presented separately.

Recent research has shown links between ADHD and restless legs syndrome (Cortese *et al*., 2005; Konofal and Cortese, 2005). As this is a relatively recent finding, we did not collect data on this in our study. This may, however, be a potential area for further investigation by others.

In a recent review paper, Corkum *et al*. (2011) highlighted the importance of assessment and treatment of sleep issues in children with ADHD. We have demonstrated on subjective measures used here that the sleep disturbances begin in infancy and that significant reductions in sleep duration at most ages predict an increased risk of ADHD. Sleep duration appears to be another potential endophenotype for ADHD that might help in both early identification for potential interventions, and also to better understand possible mechanisms. Clinically we feel there are enough normative data to allow developmental paediatric clinics to usefully collect and track sleep trajectories in the same way as other growth and physical parameters. Significant and consistent perturbations for an individual should be carefully considered in the overall developmental context for that child. We do not feel the magnitude of difference in total sleep time we determined is enough to be considered causal in the evolution of the ADHD, although we acknowledge the growing body of evidence suggesting that smaller increase in total sleep time may be worthwhile over cumulative nights (Jan *et al*., 2010). Our preference is to speculate that these early sleep peturbations are subtle early markers in some children that reflect a shared underlying pathophysiology between sleep and ADHD.
